# Development and Assessment of a Multipurpose Herbal Cream With Moringa oleifera Lam

**DOI:** 10.7759/cureus.69982

**Published:** 2024-09-23

**Authors:** Mythili Suresh, Sri Kessav Mukundan, Sivaraman Rajasekar, Sangavi Gokulakrishnan, Navesh Purushothaman, Sakthi Priyadarsini Sethuraman

**Affiliations:** 1 Department of Pharmacognosy, SRM College of Pharmacy, SRM Institute of Science and Technology, Chennai, IND

**Keywords:** anti-inflammatory, herbal cream, moringa oleifera, multipurpose cream, seed kernel

## Abstract

Background: *Moringa oleifera* Lam. (Moringaceae) is an indigenous tree extensively used as a nutrient and an antioxidant. In addition to its medicinal attributes, it possesses a promising role in moisturizing and cleansing the skin.

Objectives: Our study aims to formulate an herbal cream from *Moringa* oil and evaluate its antimicrobial and anti-inflammatory activity.

Materials and methods: In the present study, *Moringa *oil was extracted from the seed kernels and formulated into an herbal cream, and the antimicrobial and anti-inflammatory activity was evaluated. Among the three formulations M1, M2, and M3, the formulation M1 was homogenous, more stable, and non-greasy with constant pH. M1 was subjected to further antimicrobial studies and anti-inflammatory studies by the agar-well diffusion technique and albumin denaturation assays, respectively.

Results: *M. oleifera* seed kernel oil cream exhibited the highest antibacterial effect against *Staphylococcus aureus* and *Escherichia coli, *and the results were compared to the standard gentamycin. Moreover, M1 showed remarkable anti-inflammatory activity with an IC_50 _value of 124.5 μg/mL.

Conclusion: The results show that the formulated cream will be efficient in its multipurpose activity. The current study validates the antibacterial and anti-inflammatory potential of *M. oleifera* seed kernel oil cream.

## Introduction

*Moringa oleifera* is a fast-growing tree of the family Moringaceae. The plant thrives in areas located 10° and below the equator where the lands are drought-ridden. *M. oleifera* can be considered to be the world's best-kept nutritional secret [1]. The plant contains a high concentration of antioxidants, anti-inflammatory, anti-bacterial bioactives, and other phytochemicals [2].

*Moringa* seeds are rich in protein, sterols, tocopherols, and monosaturated fats in addition to their high oil content. Furthermore, the antibacterial properties and therapeutic potential of *M. oleifera* are greatly enhanced by the presence of varied metabolites including terpene, steroids, and flavonoids [3]. In addition, the plant has been used for generations in treating bacterial and fungal skin problems serving as lead antimicrobials [4,5]. 

Nowadays, herbal creams, gels, and ointments are commonly used for significant semisolid herbal formulations in topical dose therapy. Accordingly, semisolid bases are combined with extracts and isolated phytocompounds from medicinal plants to formulate a topical preparation that can treat skin problems [6,7,8]. However, the anti-inflammatory properties of medicinal plants stand as an essential stage in the process of creating herbal medicines. Formulations for herbal medicines that use locally available medicinal plants are now widespread in nations like China and India [9,10]. These reports directed us to further formulate and develop a herbal multipurpose cream serving its antimicrobial and anti-inflammatory activity. In the present investigation, *Moringa* seed kernel oil was isolated and developed into a topical herbal formulation followed by the evaluation for physical appearance, pH, and spreadability. Moreover, the antimicrobial and anti-inflammatory properties were evaluated by the agar well diffusion method and inhibition of albumin denaturation method, respectively.

## Materials and methods

Collection and authentication of the plant material

Seed kernels of *M. oleifera* Lam. (Moringaceae) were collected from Pallapatti, Madurai, and authenticated by the Plant Anatomy Research Centre, Chennai (No. PARC/2019/3958). The shade-dried seeds were decorticated, and the kernels were collected and powdered.

Test organisms, culture media, chemicals, and reagents

Two pathogenic bacterial species were used: *Staphylococcus aureus* - 902 and *Escherichia coli* - 443 from MTCC, Chandihar, India. Nutrient agar medium, nutrient broth, and gentamicin were from Himedia, India.

Preparation of seed kernel oil

About 500 grams of *M. oleifera* seed kernels were coarsely grounded and extracted in a Soxhlet extractor using petroleum ether for 15 hours. The petroleum ether extract of *M. oleifera* seed kernels, upon concentration, yielded yellowish *Moringa* oil. The *Moringa* oil thus obtained was stored in an air-tight container for further studies [11].

Phytochemical analysis

Qualitative preliminary phytochemical analysis of the seed kernel oil was carried out using various chemical detecting agents, and the nature of the metabolites was recorded [12,13]. The Dragendroff’s reagent test and Meyer’s test were carried out for the presence of alkaloids, followed by Molisch’s test for carbohydrates, Liebermann-Burchard test for steroids, ferric chloride test for tannins, Biuret test for proteins, Shinoda test for flavonoids, spot test for lipids, and Foaming test for saponins. The presence of phytoconstituents in the *Moringa* oil was detected based on the change in color in the reaction system.

Formulation of herbal cream

The standard herbal cream formulation comprises an oily phase and an aqueous phase both blended at appropriate concentrations. The herbal cream was formulated in three different compositions, M1, M2, and M3 (Table 1), and was prepared and evaluated. A fixed concentration of 15% was used for the *Moringa* oil in all three formulations. To stabilize the cream, the composition of the emulsifying agents triethanolamine and stearic acid was varied. Triethanolamine acts as the primary emulsifying agent in addition to stearic acid, which serves as an emollient. The type of cream is o/w cream. Initially, the contents of different phases were heated separately, at 60℃, and mixed with continuous stirring till they reached room temperature [14,15].

**Table 1:** Composition of Moringa oleifera herbal cream (25 g)

Ingredients	Quantity in g/mL		
	M1	M2	M3
Moringa oil	3.6 ml	3.6 ml	3.6 ml
White wax	2 g	2 g	2 g
Yellow Vaseline	8 g	8 g	8 g
Stearic acid	1.5 g	1.0 g	1.2 g
Propylene glycol	8 ml	8 ml	8 ml
Triethanol amine	1 ml	0.3 ml	0.5 ml

Evaluation of *Moringa* oil cream

The formulated creams, M1, M2, and M3, were evaluated for various parameters including appearance, homogeneity, pH, type of emulsion, spreadability, solubility, diffusion and dilution, and stability test [16-18].

In-vitro anti-inflammatory studies

Protein Denaturation Assay

About 500 μL of bovine serum albumin (1%) was added to the formulation at varying doses and heated at 51°C for 20 minutes. The absorbance reading was recorded at 660 nm after cooling with acetylsalicylic acid as standard and percentage inhibition was calculated [19,20].

Antibacterial Activity of Moringa Oil Cream (M1)

Nutrient agar medium (HiMedia) was prepared and autoclaved. About 20 mL of nutrient agar medium seeded with 1 × 10⁶ CFU/mL of bacterial culture was prepared in a Petri plate. The wells were cut and the *Moringa* oil cream (M1) at various concentrations was added and incubated for 24 hours at 37°C. Furthermore, the diameter of the inhibition zone formed around the wells was measured [21,22].

Statistical analysis

Statistical analysis was performed by one-way ANOVA using GraphPad Prism version 7.03. Values were expressed as mean ± SD (n = 3). Any value of p < 0.05 was considered statistically significant.

## Results

An initial qualitative preliminary phytochemical study was carried out using distinct chemical detection agents for the detection of carbohydrates, lipids, tannins, steroids, saponins, alkaloids, terpenoids, and flavonoids. The extracted Moringa oil showed positive results for the Salkowski test and Libermann-Burchard test indicating the presence of lipids and steroids (Table 2).

**Table 2:** Phytochemical screening of the isolated Moringa oil (+) - present; (-) - absent

S. No.	Phytoconstituents	Moringa oil
1	Alkaloids	-
2	Carbohydrates	-
3	Steroids	+
4	Tannins	-
5	Proteins	-
6	Flavonoids	-
7	Saponins	-
8	Lipids	+

**Figure 1:**
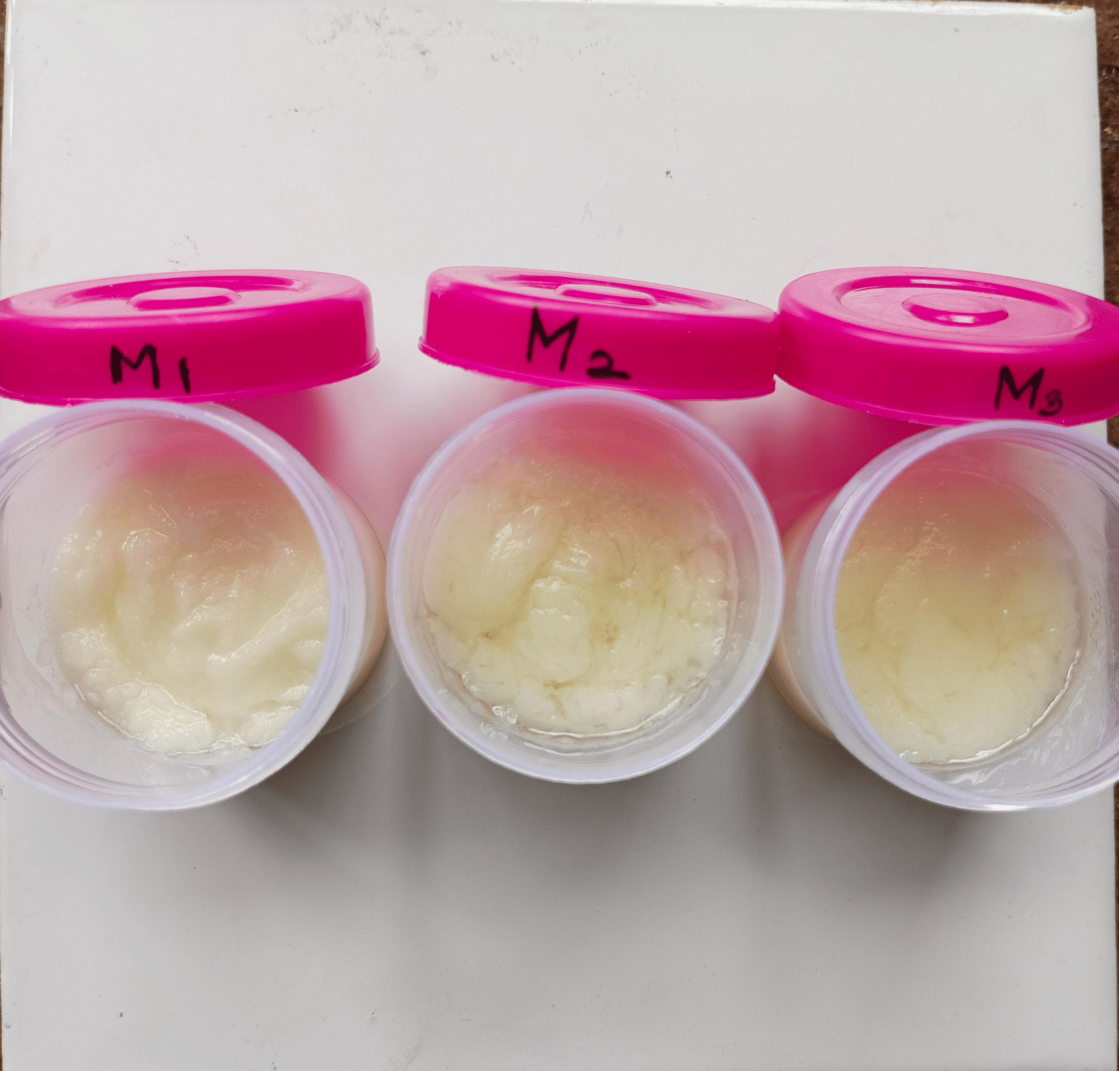
Moringa oil cream formulations: M1, M2, and M3

All three formulations were evaluated for their physical parameters (Figure 1). Among the three formulations, M1 was found to be more stable, homogeneous, emollient, and non-greasy (Table 3). There was no noticeable change in the color. All the formulations showed a pH between 5.8 and 6.8, which is closer to the skin. The viscosity ranged between 0.98 to 0.99, suggesting that it can be readily spreadable with little shear. However, compared to other formulations, M1 exhibited good spreadable properties and hence was selected for further studies. The touch and appearance, emollience, slipperiness, and spreadability are displayed in Table 3.

**Table 3:** Evaluation of the physical parameters of various formulations of Moringa oleifera cream

Parameters	M1	M2	M3
Appearance	Yellowish white	Yellowish white	Yellowish white
pH	5.8	5.6	5.7
Viscosity	0.99	0.98	0.99
Homogeneity	Homogenous	Homogenous	Homogenous
Spreadability	Easily spreadable	Easily spreadable	Easily spreadable
Dilution test	Oil in water emulsion	Oil in water emulsion	Oil in water emulsion
Emolliency	No residue	No residue	No residue
Dye solubility test	Oil in water emulsion	Oil in water emulsion	Oil in water emulsion

**Figure 2:**
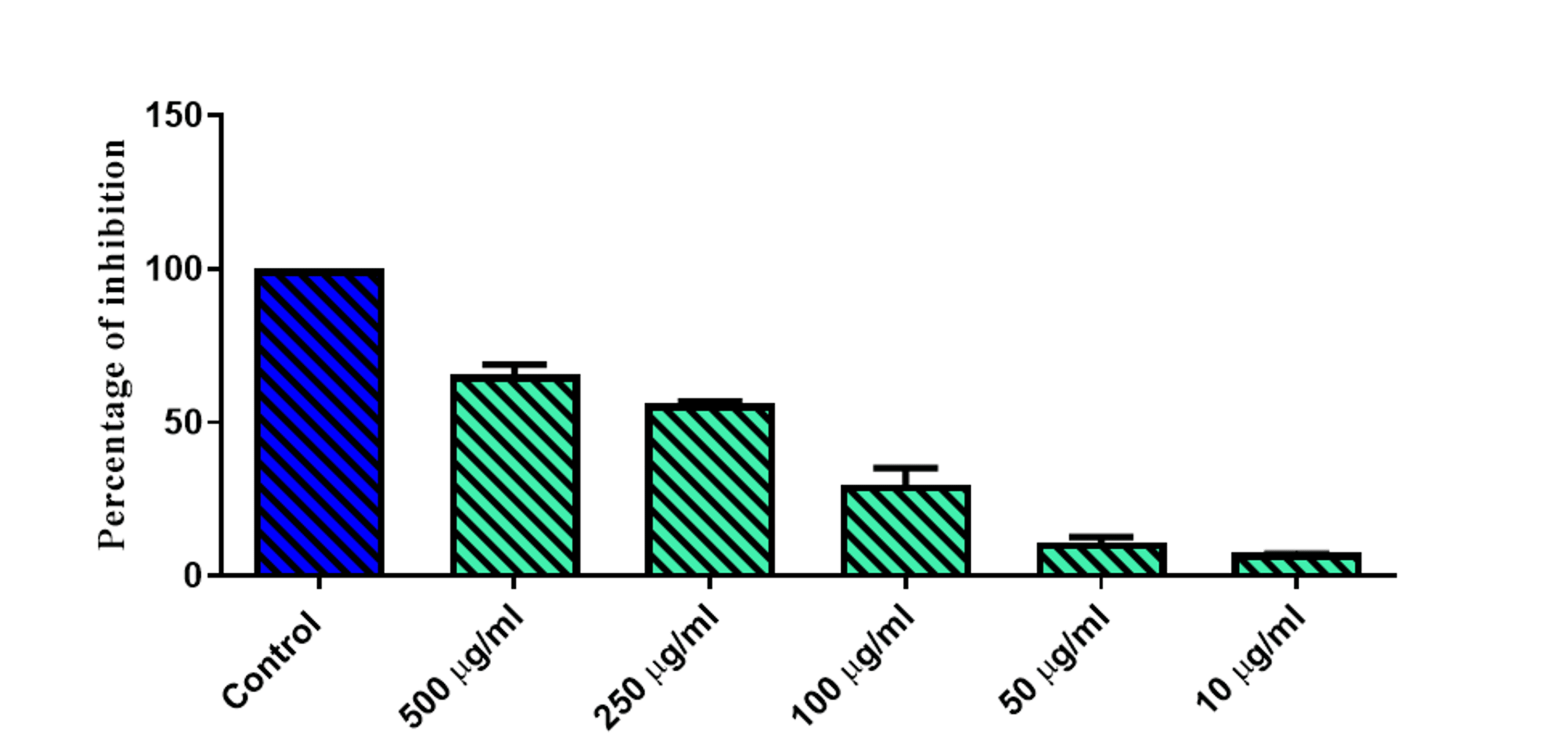
Anti-inflammatory activity of Moringa oleifera cream by the albumin denaturation assay Values expressed as mean ± SD, n = 3. Any value of p < 0.05 was considered as statistically significant.

**Figure 3:**
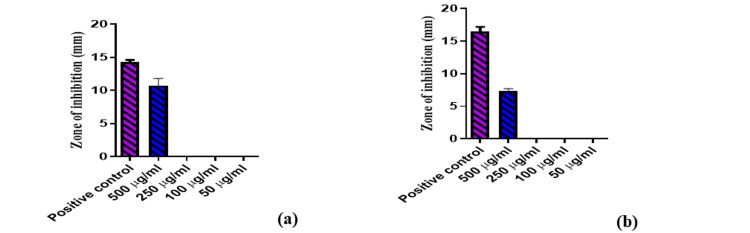
Antibacterial activity of Moringa oleifera seed kernel oil cream: Escherichia coli (a), Staphylococcus aureus (b) Values expressed as mean ± SD, n = 3. Any value of p < 0.05 was considered statistically significant.

*M. oleifera* cream showed remarkable anti-inflammatory activity with an IC50 of 124.5 μg/mL. Also, M1 exhibited the highest antibacterial effect against *S. aureus* and *E. coli*, and the results were compared to the standard gentamycin and are displayed in Figure 2 and Figure 3.

## Discussion

Secondary metabolites, found in aromatic and medicinal plants, are valuable resources with a range of uses in the pharmacological, cosmetic, and control of plant and human illnesses [23]. As a natural defensive response to tissue damage brought on by hazardous chemicals and physical trauma, inflammation occurs. Non-steroidal anti-inflammatory drugs (NSAIDs) are the most often prescribed for inflammatory diseases. NSAIDs have several side effects, including the occurrence of stomach irritation and ulcer development. According to Chandra et al. (2012), plants' abundant diversity contains new chemicals with strong anti-inflammatory properties [24].

Xu et al. (2019) conducted a comprehensive comparison of the ethanolic extracts of *M. oleifera* leaf, seed, and root from Kenya in terms of their antioxidant and anti-inflammatory potential. In addition, the authors have established a correlation between the differences in these activities and the chemical components of the plant parts. The findings of the study showed that *M. oleifera *leaves are rich in phenolic and flavonoid compounds, which may account for their superior potential for anti-inflammatory and antioxidant effects than root and seed. In addition, the flavonoid content showed a positive correlation with their antioxidant and anti-inflammatory activities [25].

Furthermore, a study by Fard et al. (2015) showed that the hydroethanolic extract of *M. oleifera *leaf had a promising anti-inflammatory effect on lipopolysaccharide (LPS)-induced macrophage inflammation. In addition, it decreased the generation of pro-inflammatory cytokines in macrophages stimulated by LPS and raised the level of IL-10 by suppressing signaling cascades that activate NFκB-p65 [26].

In a study by Saleem et al. (2020), the anti-inflammatory activity of *M. oleifera *leaves was assessed using membrane stabilization, protein denaturation, and anti-proteinase assay followed by in-vivo evaluation using a formaldehyde-induced arthritis model in Wistar rats. The results revealed that the extracts showed higher invitro antioxidant activity with a reduction in protein denaturation with a significant anti-inflammatory effect in paw and also prevented arthritis-associated anemia in a dose dose-dependent manner [10].

Sugiharto et al. recorded the anti-inflammatory activity of *M. oleifera* leaves upon *P. gingivalis*-induced inflammation. The results revealed that *M. oleifera *extract reduced the generation of cytokine IL-6 in patients with periodontitis [27]. 

Furthermore, *Moringa* is known for its culinary usage and has been an acceptable leafy vegetable. *M. oleifera* seed oil is also known for its beneficial properties, including moisturizing, anti-inflammatory, and antioxidant effects. It is commonly used in skincare products and is generally well-tolerated by most skin types. In the present study, about 15% concentration of *Moringa* oil (3.6 mL *Moringa* oil in 25 g cream) was used. This is likely safe for human application, due to its known safety profile and common folklore use. 

Furthermore, we investigated the anti-inflammatory and antimicrobial potential of our formulated *Moringa* seed kernel oil cream M1. The results of the initial phytochemical screening, which are displayed in Table 2, indicate that the *Moringa* oil contains lipids and steroids. Of the three formulations, M1 was the most homogenous, emollient, stable, and non-greasy and was selected for further antimicrobial and anti-inflammatory studies.

The efficacy of *Moringa* oil as an anti-inflammatory agent is well-supported by its composition, rich in bioactive compounds like oleic acid, tocopherols, phenolics, and flavonoids. These constituents act through various mechanisms, including antioxidant activity, inhibition of pro-inflammatory mediators, and modulation of molecular pathways like NF-κB, COX, and LOX. When applied topically, *Moringa* oil not only reduces inflammation but also promotes skin health and repair, making it a valuable ingredient in therapeutic formulations like anti-inflammatory herbal creams.

The M1 formulation showed a remarkable anti-inflammatory activity (IC50 value = 124.5 μg/mL) compared to standard acetylsalicylic acid. Furthermore, M1 exhibited the highest antibacterial effect against *S. aureus* and *E. coli*, and the results were compared to the standard gentamycin. At the maximum concentration of 500 µg/mL, the zone of inhibition of *Moringa* oil herbal cream was found to be 7.3 ± 0.42 mm and 10.75 ± 1.06 mm with a minimum inhibitory concentration (MIC) value of 5.05 × 109 cfu/ml and 1.86 × 109 cfu/ml against *S. aureus* and *E. coli*, respectively. 

However, proper preclinical experiments are necessary so that the current study can substantiate its findings with in vivo data, thereby providing a more robust validation of the herbal cream's safety, stability, and efficacy before advancing to clinical trials or consumer applications. Furthermore, experimental randomized control trials (RCTs) are required to demonstrate clinical evidence to the scientific community.

## Conclusions

The potential of plant extracts for cosmetic purposes is the main focus of the current study. The personal healthcare sector has seen an exponential increase in the field of cosmetics. The application of bioactive components in cosmetics has an impact on the skin supplying the nutrients required for healthy skin. The developed formulation contained higher consistency and better spreadability, with nil signs of phase separation possessing anti-inflammatory and antibacterial effects.

Thus, we believe that the current study validates the antibacterial and anti-inflammatory potential of *M. oleifera *seed kernel oil cream and further research will be conducted to determine the active phytocompounds responsible for the activity.
